# Polishing the tarnished silver bullet: the quest for new antibiotics

**DOI:** 10.1042/EBC20160077

**Published:** 2017-03-03

**Authors:** Mark A.T. Blaskovich, Mark S. Butler, Matthew A. Cooper

**Affiliations:** Institute for Molecular Bioscience, The University of Queensland, St. Lucia, Brisbane 4072, Australia

**Keywords:** antibiotics, antibiotic resistance, bacterial infections, drug discovery and design, Gram negative bacteria, Gram positive bacteria

## Abstract

We are facing a potential catastrophe of untreatable bacterial infections, driven by the inexorable rise of extensively drug-resistant bacteria, coupled with a market failure of pharmaceutical and biotech companies to deliver new therapeutic options. While global recognition of the problem is finally apparent, solutions are still a long way from being implemented. In addition to drug stewardship programmes and better diagnostics, new antibiotics are desperately needed. The question remains as to how to achieve this goal. This review will examine the different strategies being applied to discover new antibiotics.

## Introduction – antibiotics and antimicrobial resistance

Antibiotics have saved more lives than any other class of drugs. Yet they are misused, undervalued and now endangered by the rise of extremely resistant bacteria that cannot be treated with our current apothecary. Resistance and antibiotic misuse is nothing new – in his Nobel Prize lecture in 1945 (awarded for the discovery of penicillin), Fleming said: “It is not difficult to make microbes resistant to penicillin in the laboratory by exposing them to concentrations not sufficient to kill them, and the same thing has occasionally happened in the body. The time may come when penicillin can be bought by anyone in the shops. Then there is the danger that the ignorant man may easily underdose himself and by exposing his microbes to non-lethal quantities of the drug make them resistant” [1]. Since the introduction of the first antibiotic, there has been a constant arms race between scientists developing new and improved versions of antibiotics and bacteria developing new and improved resistance mechanisms. Because most antibiotics are derived from natural products, where they are produced as natural antibacterial defence mechanisms, resistance at some level often already exists. For example genes coding for resistance against vancomycin were found in bacterial samples isolated from 10,000-year-old permafrost [2]. The introduction of a new antibiotic is always followed by reports of clinical resistance – generally within a few years, though in a few rare cases, some antibiotic classes have lasted for decades before significant resistance has developed [3]. Just to be clear, antibiotic resistance means the bacteria have become resistant to the antibiotic (i.e. the antibiotic is no longer effective at killing the bacteria), not the human.

So what has changed to make antimicrobial resistance such a threat? We are no longer discovering and developing enough new antibiotics. In particular, we have no new antibiotics to treat drug-resistant Gram-negative bacteria, a class of microorganisms with an additional outer membrane that is difficult to penetrate and efflux pumps that prevent antibiotics from reaching concentrations required to inhibit intracellular targets. We are also abusing our existing antibiotics – approximately two-thirds of antibiotics are used on animals, not humans and much of this use is at the subtherapeutic levels as ‘growth promoters’ [4]. Of the remaining human use, another two-thirds is wasted on inappropriate prescriptions for non-bacterial infections, often given for viral infections [4]. A study in Australia has shown that almost half of the population has taken an antibiotic in the past year [5]. This misuse fosters the growth of resistance by exposing bacteria in multiple types of environments to sublethal levels of antibiotics, so that a resistant subpopulation is selected and can thrive. Genetically encoded resistance elements can then be rapidly transmitted among different types of bacteria through mobile plasmid elements. Many antibiotics have potentially toxic side effects, so the therapeutic levels used to kill normal bacteria in humans are often already close to the highest safe level possible. Even a slight reduction in the susceptibility of the bacteria to killing means the antibiotic no longer works – you cannot just increase the dose.

## Lack of new antibiotics

Why are few new antibiotics being produced? The answer really comes down to economics. Large pharmaceutical companies are essentially only interested in ‘blockbuster’ drugs with >$1 billion in annual sales. Most bacterial infections can still be treated with generic antibiotics costing only hundreds of dollars for a 1- to 2-week treatment that cures the disease – and often saves a life. Premium antibiotics designed to treat resistant infections are unable to charge more than a few thousands of dollars for a course of treatment. This is in sharp contrast with, for example, anticancer therapies that can charge over $US100,000 per year, even though they may only extend a life by months. Similarly, drugs such as those used for high cholesterol levels, which are taken continuously for years by a large population, are much more profitable. The cost of development for all three classes is similar, so it makes little financial sense for a pharmaceutical company to focus research on new antibiotics. This discrepancy is highlighted by the development pipeline: as of March 2015 there were only 28 antibiotics in Phase II/III clinical testing [6], compared with 504 oncology drugs [7].

It is not that the companies are not interested in antibacterial therapies – the Prevnar vaccine to prevent *Streptococcus pneumoniae* infections had $6.2 billion in sales in 2015 [8], explaining why a number of companies are pursuing vaccines, compared with relatively few still developing antibiotics. From approximately 20 traditional pharmaceutical companies investing in antibiotic research in the 1980s, there are now only approximately five with significant active internal research programmes: Novartis, GSK, Roche, Sanofi and Merck (which acquired Cubist, a highly successful antibiotic company, in 2014, but then disbanded the Cubist research staff, while retaining its own antibacterial division). Until recently AstraZeneca was also on this list, but it spun out its discovery group into Entasis Therapeutics in mid-2015, with the small company retaining only 21 of AstraZeneca’s 175 antibiotic researchers [9] and in 2016 sold its advanced antibiotic products to Pfizer. The departure of thousands of antibiotic researchers from major pharmaceutical companies over the past two decades has led to an immeasurable loss of collective wisdom in antibiotic development, a specialized discipline different from other therapeutics. Fortunately, some of this knowledge is being transferred to smaller companies, as exemplified by several recent startups (Kaleido Biosciences and Spero Therapeutics), fostered in part by the layoff of Cubist research staff after its acquisition by Merck [10].

Most novel antibiotic development is arguably now being conducted at similar small biotech companies – they are able to make a financial case that an antibiotic with a much smaller potential market (e.g. $200 million/year) will provide a return on investment. Over 40 antimicrobial focused biotech companies are a part of the BEAM alliance (Biotechs from Europe innovating in Anti-Microbial Resistance) [11]. However, once these small companies have done the ground work of antibiotic discovery, partnerships with traditional pharma companies are usually needed to either fund the expensive late stage clinical trials needed to gain approval or to conduct the marketing campaign once an antibiotic is approved. This explains why, for example Roche has partnered with a number of small biotech antibiotic companies, including Polyphor (2013), RQx (2013), Spero Therapeutics (2014), Discuva Ltd (2014), GeneWeave (2015), Meiji Seika Pharma and Fedora (2015). The interest from large pharma companies extends to support antibiotic-focused biotechs: for example the venture capital arms of Novartis, Roche and GlaxoSmithKline all invested in Macrolide Pharmaceuticals, a new company founded in 2015 by Andrew Myers. Myers previously created Tetraphase Pharmaceuticals in 2006, focused on tetracycline antibiotics, though its lead candidate eravacycline failed a Phase III trial in 2015.

The dire strait of antibiotic research has increasingly been recognized by national and international agencies, with numerous reports published over the past few years, including from the World Health Organization [12,13], the G7 [14], and national governments of the United Kingdom [15–17], the United States of America [18–22], Canada [23,24], Australia [25], Sweden [26], Germany [27] and Norway [28]. The U.K. Prime Minister commissioned a Review on Antimicrobial Resistance, chaired by economist Jim O’Neill, which has produced a comprehensive series of reports: ‘Antimicrobial resistance: tackling a crisis for the future health and wealth of nations’ (Dec 2014) [29], ‘Tackling a global health crisis: initial steps’ (February 2015) [17], Securing new drugs for future generations – the pipeline of antibiotics’ (May 2015) [30], ‘Rapid diagnostics: stopping unnecessary use of antibiotics’ (October 2015) [31], ‘Safe, secure and controlled: managing the supply chain of antimicrobials’ (November 2015) [32], ‘Antimicrobials in agriculture and the environment: reducing unnecessary use and waste’ (December 2015) [33], ‘Vaccines and alternative approaches: reducing our dependence on antimicrobials’ (February 2016) [34], ‘Infection prevention, control and surveillance: limiting the development and spread of drug resistance’ (March 2016) [35] and ‘Tackling drug-resistant infections globally: final report and recommendations’ (May 2016) [36]. The potential threat posed by a pandemic of extremely drug-resistant (XDR) bacteria means that some governments are starting to include these scenarios in natural disaster planning [37]. On September 21, 2016, the United Nations General Assembly held a ‘high-level meeting’, in which representatives of 70 governments discussed controlling resistance, only the fourth time that the General Assembly had ever considered a health problem. The result was a commitment by all 193 member nations to begin working on the problem, starting with a ‘co-ordination group’ to harmonize current international efforts.

A large range of academic reviews and discussions on the antibiotic crisis have been recently published. These include articles on the antibiotic crisis [38–41] and possible solutions [42,43], sources of resistance [44], surveillance of resistance [45], restricting non-medical antibiotic use [46], antibiotic resistance in livestock and the environment [47,48], possible approaches to address R&D and commercialization challenges [49–51], difficulties in discovering new antibiotics [52], development of new antibiotics [53–55], reviews of therapeutic strategies [56] and new approaches to discover novel antimicrobials [57,58] to combat antibiotic resistance.

The problem is, all these reviews and discussion papers identify the serious issues posed by antimicrobial resistance and propose potential solutions and pathways to address the problems, such as methods to incentivize pharmaceutical companies to invest in research, but implementation of these policies is a long way in the future and, to date, little practical action has been taken.

## New antibiotic discovery

**Figure 1:**
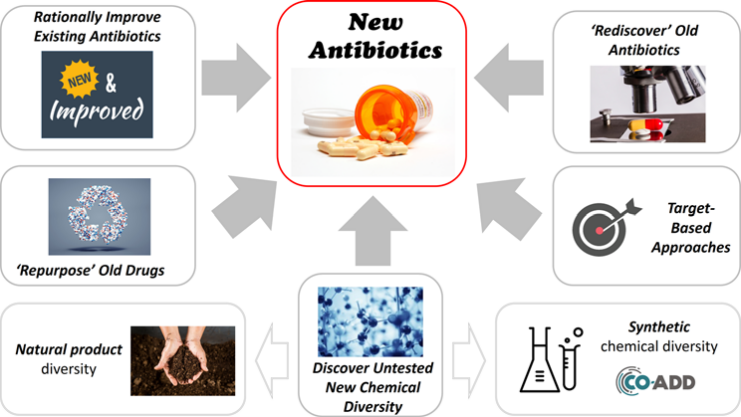
Different approaches to discover new antibiotics

So, how do we discover new antibiotics? There are a number of different possible approaches (see Figure 1).

### Rationally improve existing antibiotics

Most new antibiotics are improvements of existing classes – you take an existing antibiotic and try to improve its potency or make modifications to overcome resistance. This is why we have fifth or sixth generation versions of penicillin, one of the original antibiotics. This approach is illustrated by a project within the Centre for Superbug Solutions (CSS) at the University of Queensland, where we have developed an improved version of vancomycin by chemically attaching substituents that selectively target vancomycin to the surface of bacteria in preference to human cells. By doing this, we increase its potency and reduce its toxic side effects [59].

### ‘Rediscover’ old antibiotics

**Figure 2:**
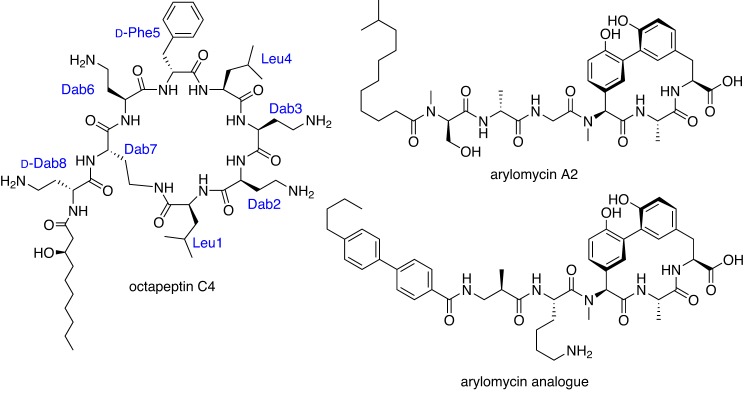
Examples of ‘rediscovered’ antibiotics

Hundreds, if not thousands, of antibiotics were reported in the literature during the ‘Golden Age’ of antibiotic research, the 1950s–1960s. Only some of these were advanced into human use due to a plethora of choices at the time. By mining the literature, we can potentially uncover these hidden gems, identifying overlooked antibiotics that could now be resurrected and developed into drugs. An example of this approach is again shown by researchers at CSS, who are investigating a class of antibiotics related to an antibiotic of last resort, colistin. Colistin was first developed in the 1950s, but was rarely used in humans due to the toxic side effects. It is now the only antibiotic that still works against some XDR bacteria, but unfortunately resistance to colistin has begun to develop very quickly [60]. Several publications from the 1970s described a related class of compounds, octapeptins (e.g. octapeptin C4, Figure 2) [61] and hinted that they worked against bacteria that had developed resistance to colistin. We have now made over 300 analogues of these compounds and found some that look very promising for further development (Gallardo-Godoy, A., Swarbrick, J.D., Blaskovich, M.A.T., Elliott, A.G., Han, M., Thompson, P.E. et al., unpublished work).

A more recent ‘rediscovery’ example is provided by the arylomycins (e.g. arylomycin A_2_ and arylomycin analogue, Figure 2), Gram-positive antibiotics that were first reported from *Streptomyces* sp. Tü 6075 in 2002 [62,63] and were later shown to inhibit type I signal peptidase (SPase) [64–66]. SPase is a membrane-bound serine endopeptidase that catalyses the cleavage of the N-terminal signal peptide from secretory and membrane proteins and has been seen as a promising target as it is essential for bacterial viability and growth [67]. The Romesberg Group at the Scripps Research Institute has undertaken a series of structural modifications that have formed the basis of RQx Pharmaceuticals [68–71]. In 2011, Roche (Genentech) licensed RQx’s arylomycin program. Although there have been no further development updates, Genentech recently reported that an ABC transporter was up-regulated *in vitro* to circumvent inhibition of SPase but not *in vivo* [72].

### ‘Repurpose’ old drugs

A number of clinically used drugs that were initially approved for non-infectious disease purposes or for one type of pathogen, have since been redeveloped as new antibacterials with a novel mechanism of action. This approach has the advantage that safety and development pathways are already established [73,74]. For example in the 1970s and early 1980s, Hoechst A.G. discovered and developed fexinidazole through preclinical development as a broad-spectrum antimicrobial agent, but further development was not pursued. In 2005, the Drugs for Neglected Diseases initiative (DNDi) identified fexinidazole as a candidate for treating human African trypanosomiasis (HAT, also known as sleeping sickness) [75] and it is currently undergoing a Phase III clinical trial. In 2012, the National Center for Advancing Translational Sciences (NCATS) at the U.S. National Institutes of Health (NIH) announced a drug repurposing initiative, ‘Discovering New Therapeutic Uses for Existing Molecules’ (New Therapeutic Uses) (www.ncats.nih.gov/ntu), with a new phase launched in 2014 to make 26 abandoned drugs from pharmaceutical companies such as AstraZeneca, Janssen Research & Development, Pfizer and Sanofi available to academic researchers, along with grant funding. To date, the funded projects have not included infectious diseases. In addition, companies such as Helperby Therapeutics Ltd (London) and organizations such as Antibiotic Resistance U.K. (ANTRUK) are investigating possible synergistic interactions of marketed drugs as ‘resistance breakers’ when used in combination with the current antibiotics [76].

### Discover untested new chemical diversity

The past decade has seen a concerted effort to return to natural product discovery – the original source of most existing antibiotics. But, to prevent simply rediscovering the same antibiotics, there are new variations in how this is being done [77].

1. Investigate new sources of the bacteria or fungi e.g. extremophiles that grow in thermal vents, marine organisms or microorganisms in Antarctica. This approach is being used by the Marine Bioproducts Engineering Center, a partnership between the University of Hawaii at Manoa and the University of California at Berkeley [78].
2. Soil samples contain hundreds if not thousands of microbes, but in the past we have only been able to grow a small percentage of these. We can uncover new bacteria/fungi and their associated metabolites, by culturing them under a range of conditions where more can grow, as was recently reported for the discovery of a new antibiotic, teixobactin [79], where NovoBiotic Pharmaceuticals (Cambridge, MA) used an ‘iChip’ to culture and isolate bacteria *in situ* in soil.
3. Force microorganisms to produce different metabolites by growing them under different conditions i.e. at different temperatures, with different growth media [80]. In 2014, Sanofi partnered with the Fraunhofer Institute for Molecular Biology and Applied Ecology (Aachen, Germany) in a joint effort to explore natural products, mining Sanofi’s collection of >100000 different microorganisms to cultivate them under various conditions and stimulate the production of active substances [81].
4. Look for metabolites at the genetic level by conducting microbiome whole-genome sequencing to identify gene clusters predicted to produce metabolites [80,82,83]. Warp Drive Bio LLC (Cambridge, MA), Verenium Corporation (San Diego, CA; previously Diversa) and the former TerraGen Discovery Inc. (taken over by Cubist in 2000) are utilizing genomic-screening technologies to look in gene expression libraries from DNA extracted from environmental samples, searching for specific sequences that may be manipulated to produce novel antimicrobials. For example a novel cyclodepsipeptide, named NC-1, was identified by genomic sequencing of red soil-derived *Streptomyces* sp., then activated and isolated and found to have antimicrobial activity [84]. Ribosomally produced, post-translationally modified products can also be predicted and isolated [82].
5. Find new synthetic chemical diversity – instead of trying to isolate new antibiotics from the pool of natural products, mine the cumulative collection of synthesized compounds.

### Crowdsourcing new antibiotics

**Figure 3:**
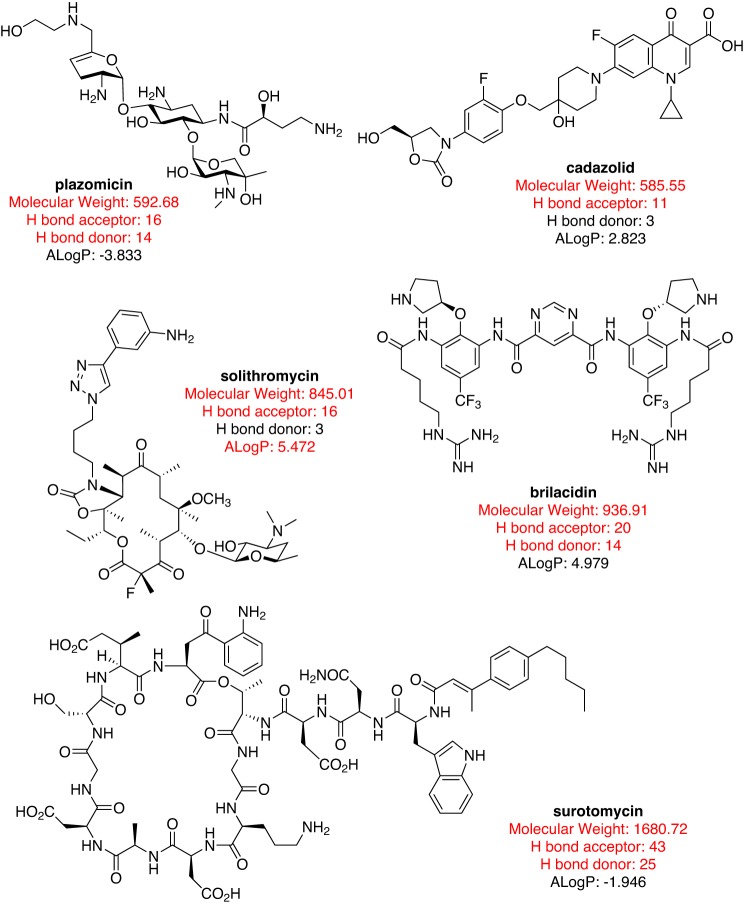
Approved or late-stage clinical antibiotics that do not obey drug-like rules for oral availability (molecular weight ≤500, H-bond acceptor ≤5, H-bond donor ≤ 5 and log *P*≤5)

**Figure 4:**
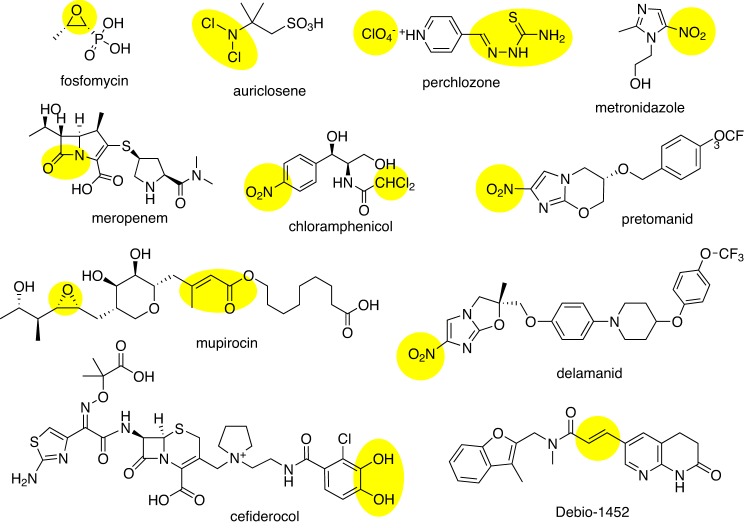
Approved or late-stage clinical antibiotics with ‘undesirable’ chemical functional groups

CO-ADD, the Community for Open Antimicrobial Drug Discovery (www.co-add.org), is taking this last approach, searching for new chemical diversity by crowdsourcing compounds from academic chemists around the world [85–87]. The premise of CO-ADD lies in an assumption that one reason antibiotic discovery has been recently so unproductive is that pharmaceutical compound collections are now curated with an emphasis on compounds with ‘drug-like’ properties (often relating to oral availability), removing compounds that have ‘undesirable’ substituents or which do not comply with certain physico-chemical rules. However, antibiotics are different from most other drugs. Because of their natural product origin and acceptability for intravenous dosing, many antibiotic classes fail to comply with almost all of the ‘drug-like’ rules, such as Lipinski’s ‘Rule of five’ or number of rotatable bonds (Figure 2 in [85] and Figure 3). Furthermore, many marketed antibiotics contain functional groups (Figure 4) that if a modern medicinal chemist presented as part of a new molecule selected for hit to lead development in a standard drug-development programme, would not be viewed favourably by his peers.

**Figure 5:**
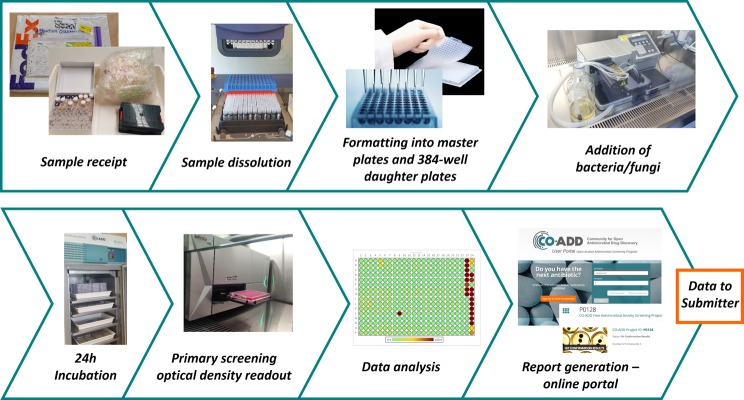
The CO-ADD screening workflow

CO-ADD is returning to another aspect of how antibiotics were originally discovered – through collaboration. Chemists and biologists from industry and academia used to work together by sending compounds back and forth, without legal agreements or barriers to collaboration, because they were focused on trying to stop people dying from infections. CO-ADD is attempting to recreate this collaborative effort by fostering a co-operative environment with as low a barrier to participation as possible. Importantly, all the rights to the compounds and data are kept by the chemist sending the compound. CO-ADD has been incredibly successful – in just over 18 months over 200 academic groups in 35 countries have sent more than 120,000 compounds. CO-ADD is building the world’s largest, most diverse collection of compounds ever assembled. CO-ADD conducts an initial single concentration screen against five bacteria and two fungi, with ‘hits’ undergoing a hit confirmation dose–response assay and counter screening for toxicity against mammalian cells (see Figure 5). CO-ADD has identified hundreds of active compounds – but it is still too early to say whether any of them have the potential to become a life-saving antibiotic. The package of data produced by CO-ADD matches the entry information required by organizations focused on providing funding and support to transition well-characterized hit molecules into antibiotic drug development programmes – namely the IMI ENABLE consortium (Innovative Medicines Initiative European Gram-negative Antibacterial Engine, part of the New Drugs for Bad Bugs (ND4BB) programme) (see www.imi.europa.eu/content/enable), and the more recent CARB-X initiative (see www.carb-x.org/). IMI ENABLE is an €85 million project that seeks promising early stage Gram-negative programmes from European small and medium-sized enterprises (SMEs) or research groups, with the mission to bring at least one candidate to Phase I clinical trial via an international consortium of partners. CARB-X is a global antibacterial public–private partnership resulting from the 2015 U.S. National Action Plan on Combating Antibiotic-Resistant Bacteria that aims to leverage $US250 million in BARDA funds with matching funds from the Wellcome Trust and the AMR Centre (U.K.) in order to accelerate the development of at least 20 high quality antibacterial products towards clinical development.

### Target-based approaches

As with large pharma screening their commercial libraries, the rational drug design approach, based on identifying bacterial-specific targets, has so far proven to be spectacularly unsuccessful. On the surface, this approach has great potential – whole genome sequencing can help to identify unique targets specific to bacteria, providing high specificity over human cells, and targets can be selected that are either common to all bacteria or specific to certain species. In reality, a number of promising targets have been identified and highly specific and potent inhibitors developed. However, as soon as these are applied to kill or inhibit the whole organism, the multiple defence mechanisms of bacteria take over, preventing entry, actively exporting or quickly mutating the target. In particular, the traditionally preferred enzymatic targets of structure based design programmes, with a well-defined binding pocket, are often susceptible to single-point mutations that rapidly confer resistance – much more rapidly than seen in human-targeted therapeutics due to the rapid division of bacterial cells (often 20 min) and a large number of cells present in an infection (potentially 10^5^ to 10^9^ cfu/ml in an infected site). Both GSK [88] and AstraZeneca [52] have reported the limitations of target-based screening within their organizations.

**Figure 6:**
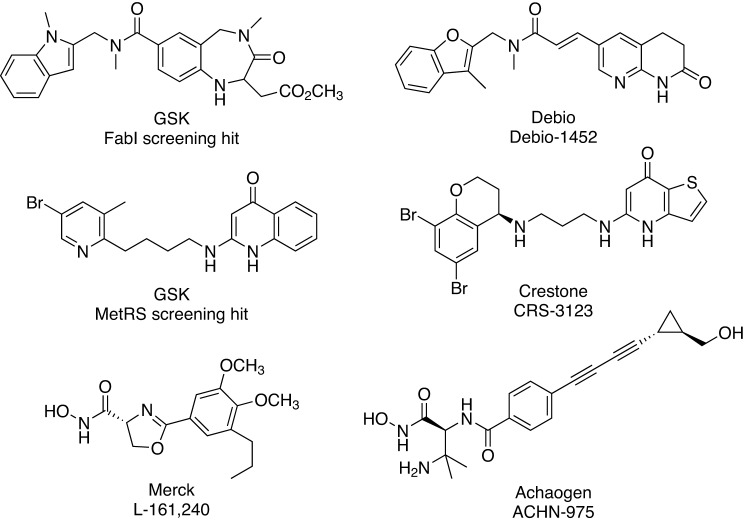
Target-based antibiotics

There are only a limited number of compounds derived from target-based approaches that have reached clinical trials (Figure 6), including Debio-1452 (previously AFN-1252, targets FabI) and CRS3123 (previously REP-3123, targets MetRS), which have been previously reviewed [88,89] and ACHN-975, which is an inhibitor of UDP-3-*O*-(3*R*-hydroxyacyl)-*N*-acetylglucosamine deacetylase (LpxC). LpxC is a Zn^2+^-dependent enzyme that catalyses the first step in the biosynthesis of Lipid A, an integral part of the lipopolysaccharide (LPS) that acts a hydrophobic membrane anchor and is essential for cell viability [90]. The first LpxC inhibitors, containing a hydroxamic acid moiety that binds to the Zn^2+^, were reported by Merck & Co. in 1996 [91]. Although there have been many hydroxamic acid-containing inhibitors synthesized and evaluated for their antibacterial activity [90], the first to enter clinical trials was Achaogen’s ACHN-975 [92]. ACHN-975 completed one Phase I trial (NCT01597947) but a second trial (NCT01870245) was terminated, reportedly due to injection site problems [90]. Achaogen is actively working on second-generation analogues that are currently undergoing preclinical evaluation with support from a NIAID contract awarded in 2015 [93].

## Conclusions

**Figure 7:**
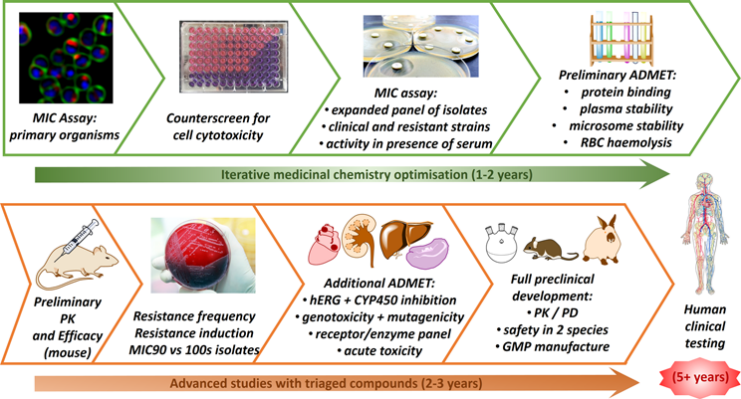
Typical antibiotic drug development pathway

The problem with antibiotic drug discovery is that it takes a long time and success rates are lower than that for other drugs. For a compound with promising activity that was discovered in a test tube today, it would take a couple of years of research to find a candidate suitable for further development as a drug and then another couple of years (and several million dollars) to complete all the testing required to undertake a first trial in humans. If the project is lucky, the three stages of human clinical trials needed to get a drug approved may be completed within five years, but existing antibiotics have often taken more than 10 years (see Figure 7). So, from initial identification of a promising new compound, it will be 10–15 years before the new antibiotic is available to be used to treat bacterial infections in humans. Organizations such as IMI ENABLE and CARB-X have been established to help accelerate this process, by providing antibiotic development expertise and funding to help bridge the gap between early discovery compounds and clinical development.

A world without antibiotics is hard to imagine. Routine surgeries – things like hip and knee replacements – would no longer be as safe as they are today. Organ transplants would be risky and cancer therapies would be compromised, as the accompanying immunosuppression would rapidly lead to bacterial and fungal infections. Our current antibiotics work now, for the most part. But, if we get an epidemic of one of the XDR strains rapidly spreading across the globe, it will be too late to start funding the discovery of new antibiotics. We need to invest now, so we have options when the inevitable next ‘black death’ appears.

## Summary

- The world urgently needs new antibiotics.
- Antimicrobial resistance is rapidly increasing and spreading, but a few large pharmaceutical companies are investing into antibiotic research.
- New antibiotics can be discovered by rationally improving existing antibiotics, rediscovering old antibiotics, repurposing old drugs or discovering new untested chemical diversity.
- Organizations such as CO-ADD, IMI-ENABLE and CARB-X are attempting to refill the antibiotic pipeline by assisting the discovery and development of new antibiotics.

## Abbreviations

AMR: antimicrobial resistance
BARDA: Biomedical Advanced Research and Development Authority
CO-ADD: Community for Open Antimicrobial Drug Discovery
CSS: Centre for Superbug Solutions
GSK: GlaxoSmithKline
G7: Group of 7
H-bond: hydrogen-bond
IMI ENABLE: Innovative Medicines Initiative European Gram-negative Antibacterial Engine
LpxC: UDP-3-*O*-(3*R*-hydroxyacyl)-*N*-acetylglucosamine deacetylase
NIAID: National Institute of Allergy and Infectious Diseases
R&D: research and development
SPase: signal peptidase
XDR: extremely drug-resistant
